# Mitochondrial Determinants of Anti-Cancer Drug-Induced Cardiotoxicity

**DOI:** 10.3390/biomedicines10030520

**Published:** 2022-02-22

**Authors:** Carmine Rocca, Ernestina Marianna De Francesco, Teresa Pasqua, Maria Concetta Granieri, Anna De Bartolo, Maria Eugenia Gallo Cantafio, Maria Grazia Muoio, Massimo Gentile, Antonino Neri, Tommaso Angelone, Giuseppe Viglietto, Nicola Amodio

**Affiliations:** 1 Laboratory of Cellular and Molecular Cardiovascular Pathophysiology, Department of Biology, Ecology and Earth Sciences (DiBEST), University of Calabria, Arcavacata di Rende, 87036 Cosenza, Italy; carmine.rocca@unical.it (C.R.); mariaconcetta.granieri@unical.it (M.C.G.); anna.de_bartolo@unical.it (A.D.B.); 2 Unit of Endocrinology, Department of Clinical and Experimental Medicine, University of Catania, Garibaldi-Nesima Hospital, 95122 Catania, Italy; ernestina.defrancesco@unict.it (E.M.D.F.); mariagrazia.muoio@unict.it (M.G.M.); 3 Department of Health Science, University Magna Graecia of Catanzaro, 88100 Catanzaro, Italy; teresa.pasqua@unicz.it; 4 Department of Experimental and Clinical Medicine, Magna Graecia University of Catanzaro, 88100 Catanzaro, Italy; mariaeugenia.gallocantafio@unicz.it (M.E.G.C.); viglietto@unicz.it (G.V.); 5 Hematology Unit, “Annunziata” Hospital of Cosenza, 87100 Cosenza, Italy; m.gentile@aocs.it; 6 Department of Oncology and Hemato-Oncology, University of Milan, 20122 Milan, Italy; antonino.neri@unimi.it; 7 Hematology Fondazione Cà Granda, IRCCS Policlinico, 20122 Milan, Italy; 8 National Institute of Cardiovascular Research (I.N.R.C.), 40126 Bologna, Italy

**Keywords:** anticancer therapy, cardiotoxicity, heart failure, mitochondrial function

## Abstract

Mitochondria are key organelles for the maintenance of myocardial tissue homeostasis, playing a pivotal role in adenosine triphosphate (ATP) production, calcium signaling, redox homeostasis, and thermogenesis, as well as in the regulation of crucial pathways involved in cell survival. On this basis, it is not surprising that structural and functional impairments of mitochondria can lead to contractile dysfunction, and have been widely implicated in the onset of diverse cardiovascular diseases, including ischemic cardiomyopathy, heart failure, and stroke. Several studies support mitochondrial targets as major determinants of the cardiotoxic effects triggered by an increasing number of chemotherapeutic agents used for both solid and hematological tumors. Mitochondrial toxicity induced by such anticancer therapeutics is due to different mechanisms, generally altering the mitochondrial respiratory chain, energy production, and mitochondrial dynamics, or inducing mitochondrial oxidative/nitrative stress, eventually culminating in cell death. The present review summarizes key mitochondrial processes mediating the cardiotoxic effects of anti-neoplastic drugs, with a specific focus on anthracyclines (ANTs), receptor tyrosine kinase inhibitors (RTKIs) and proteasome inhibitors (PIs).

## 1. Introduction

Despite the great energy consumption needed for contraction and ion transport, the human heart is characterized by a limited content of endogenous high-energy phosphate, able to support cardiac activity only for a very short time [1]. For this reason, adenosine triphosphate (ATP) is constantly produced, especially by mitochondria which, beside representing one third of myocyte volume, account for more than 95% of the cardiac ATP [2]. Mitochondria not only produce ATP by oxidative phosphorylation (OXPHOS), but are also involved in the balance of the redox status, in Ca^2+^ homeostasis, and in the modulation of nuclear gene expression that may result in the regulation of crucial pathways involved in cell survival [3]. Hence, it is not surprising that disorders of these organelles may disrupt cardiac physiology, leading to cardiovascular diseases (CVDs), as convincingly demonstrated by different comprehensive studies [4,5]. Over the past decades, further information has described mitochondria as dynamic organelles undergoing a finely tuned process, known as mitochondrial dynamics, which contributes to cellular homeostasis, allowing the generation of an appropriate response to environmental changes [6,7,8,9]. Moreover, to accomplish their activities, mitochondria exploit a selective quality control machinery whose purpose is to target and remove misfolded proteins or aberrant organelles which could impair cardiac homeostasis [4,10].

Because of the dominant role of mitochondria in calcium signaling, redox homeostasis, and thermogenesis, as well as in dictating the fate of a cell, mitochondrial disorders represent a major challenge in medicine [11,12]. Mitochondrial impairment—in terms of defective apoptosis, cytoplasmic and mitochondrial matrix calcium regulation, reactive oxygen species (ROS) generation and detoxification, ATP generation, metabolite synthesis, and intracellular metabolite transport—has been implicated in diverse pathological conditions. Specifically, mitochondria predominantly contribute to maintaining the heart’s homeostasis; thus, structural and functional alterations in this organelle lead to contractile dysfunction, and underlie the pathophysiology of several cardiovascular diseases (CVDs), including ischemic cardiomyopathy, heart failure, and stroke [4,13].

Recently, mitochondrial targets have also emerged as important determinants in the cardiotoxic effects triggered by an increasing number of chemotherapeutic agents [14,15], which clinically present as a dose-dependent cardiomyopathy leading to chronic heart failure (CHF), significantly impacting morbidity and mortality [16]. Given the increasing number of long-term cancer survivors and the clinical impact of chemotherapy-related cardiotoxicity, standardizing risk stratification, evaluating the multifactorial processes relying on the interaction between genetic and environmental factors during anticancer therapy, and improving the knowledge of the mechanisms underlying anticancer-drug cardiotoxicity and cardiovascular adverse effects (CVAEs) still represent major challenges in the field of cardio-oncology [13,16]. 

In this perspective, the present review aims to provide a comprehensive analysis of the key role played by mitochondria in cardiac patho-physiology, focusing on mitochondrial processes implicated in normal cardiac homeostasis, and on their perturbations upon treatment with those cardiotoxic anti-neoplastic drugs which are relevant from a cardio-oncology viewpoint, namely anthracyclines (ANTs), receptor tyrosine kinase inhibitors (RTKIs) and proteasome inhibitors (PIs). 

## 2. Mitochondria and Heart Physio-Pathology

**Energy supply in cardiac cells**. To cope with the energy demands of the heart, mitochondria produce ATP from a wide range of substrates, such as carbohydrates, fatty acids, amino acids and ketone bodies; however, under basal conditions, energy is mainly drawn from fats (60–90% of cardiac energy supply) [1]. Specifically, while fatty acids (FAs) are directly subjected to β-oxidation in the mitochondria, glucose is preliminarily subjected to glycolysis in the cytosol to produce pyruvate, which in turn is transferred to the mitochondria for oxidation. Usually, glucose and FAs establish a reciprocal relationship described by the Randle cycle, i.e., a dynamic adaptation that induces cardiomyocytes to use these energetic substrates depending on their availability [17,18]. Altered mitochondria result in impaired ATP production and defective energy metabolism that may predispose a higher risk for developing cardiac diseases [10,19].

**Redox homeostasis**. The oxidative phosphorylation that leads to ATP synthesis is accompanied by electron shift, as visible in the electron transport chain (ETC) by the contribution of electron carriers such as FADH_2_ and NADH. During this process, a small number of electrons (0.2–2%) slip and are transferred to O_2_ to form superoxide [2]. This phenomenon helps explain why mitochondria represent the main cellular source of ROS, as byproducts of electron transfer, whose accumulation not only causes mitochondrial injury but also can lead to the development of cardiovascular diseases. To regulate oxidative stress, mitochondria employ efficient networks, able to scavenge ROS [2,20], which importantly supports the general antioxidant activity of cardiac cells, mitigating oxidative stress [21,22,23]. The first defense against mitochondrial ROS is represented by superoxide dismutase (SOD), which transforms the superoxide anion into hydrogen peroxide; the latter is then detoxified by catalase, glutathione peroxidase (GSH-PX), and peroxiredoxin/thioredoxin (PRX/Trx) systems. Catalase is a crucial element of the intracellular ROS detoxification process, and is localized not only in peroxisome but also in cardiac mitochondria [24], indicating a role in controlling the ROS pool of these organelles; these enzymes act on hydrogen peroxide, generating water and oxygen. GSH-PX1 and GSH-PX4 are confined in the mitochondria and, by using reduced glutathione (GSH), convert hydrogen peroxide into water and produce oxidized glutathione (GSSG), which is next reconverted into GSH by glutathione reductase with the support of NADPH [25]. In addition, GSH represents a non-enzymatic antioxidant, able to directly neutralize the hydroxyl radical [26]. In this context, it is important to underline that the GSH/GSSG ratio can be considered a useful indicator of oxidative stress [27]. Of note, even if both catalase and GSH-PX are able to reduce hydrogen peroxide, they show important catalytic differences. GPX-PX reduces hydrogen peroxide by making use of glutathione, while catalase mainly acts through the Fenton reaction [28]. Moreover, a differential role of these enzymes in their scavenging activity has been postulated, indicating catalase as a primary defense against low hydrogen peroxide concentrations and GSH-PX as a protective system under high hydrogen peroxide levels [29]. 

**Ionic balance**. A fine regulated ion balance, obtained by the presence of selective channels and appropriate exchangers, ensures the physiological potential of the mitochondrial membrane that, in turn, contributes to correct redox regulation and ATP production. In particular, the mitochondrial membrane potential (ΔΨm) and the negative charge detectable in the matrix are generated by the flow of electrons in the respiratory chain, and act as a crucial driving force for ATP synthesis [30]. Accordingly, ΔΨm represents a useful indicator of cardiac cell health, and its preservation is vital for cardiomyocytes [31,32]. Calcium channels and transporters are localized on both the outer (OMM) and the inner (IMM) mitochondrial membranes [33,34,35] and make mitochondria able to detect calcium cytosolic signaling and eventually mediate its sequestration [36]. It is well established that the amount of intracellular calcium (100 nM) is more than 10,000-fold less than the extracellular [37], and that in the mitochondrial matrix calcium levels range from 100 to 200 nM under resting conditions [38]. When several stresses induce an increase of intracellular Ca^2+^ levels, mitochondria act as efficient Ca^2+^ buffering organelles [39]. A rise in intracellular Ca^2+^ increases mitochondrial uptake [40], causing an elevation of intra-mitochondrial Ca^2+^ and a drop in ΔΨm that enhances ROS production and oxidative stress.

**Programmed cell death**. A wide range of stimuli may activate mitochondrial-related apoptosis, as in the case of ischemia/reperfusion (I/R), loss of nutrients, oxidative stress, increased Ca^2+^ levels, chemotherapeutics, and targeted cancer therapies [41]. The main event in the mitochondria-driven apoptotic process is the permeabilization of the OMM, which allows several apoptogens to move towards the cytosol and activate procaspases. The whole mechanism is strictly regulated by the BCL-2 (B cell lymphoma-2) proteins [42], a protein family including three subfamilies, which are grouped according to their function and to the BCL-2 homology (BH) domains: (i) pro-survival proteins (containing BH1-4), such as BCLW, MCL-1, BCL-xL, and BCL-2 itself; (ii) pro-cell death proteins (containing BH1-3, or rarely BH1-4), such as BAX, BAK, and BOK; and (iii) pro-cell-death proteins (containing only BH3) such as BIM, BID, PUMA, and NOXA [41]. BH3 proteins are able to physically bind BAX and BAK, inducing their conformational activation, which results in their homo- or hetero-oligomerization within the OMM [43]. This critical step produces OMM permeabilization and the leak of apoptogens [44,45,46] from the mitochondria with the activation of cytosolic pro-caspases, which in turn trigger apoptosis [47]. In particular, the released cytochrome c induces the assembly of the apoptosome, a multiprotein complex that activates caspase-9 by the cleavage of pro-caspase-9, then inducing other apoptotic effectors [48,49,50]. Conversely, BCL-2 is able to both sequester BH-3 proteins and bind BAX/BAK, inhibiting this death process and promoting cell survival [41,51]. 

### 2.1. Mitochondrial Quality Control

Cardiac homeostasis strictly depends on healthy mitochondria, and for this reason they exploit a selective quality control machinery that, by targeting damaged mitochondria or mitochondrial proteins, drives them to degradative and/or removal processes [4]. Indeed, several cardiomyopathies are characterized by the presence of abnormal mitochondria clusters [4,10,19]. Two main pathways intervene to support the quality control of mitochondria: (i) the ubiquitin-proteasome system (UPS), which degrades damaged mitochondrial proteins; and (ii) the autophagy-lysosomal pathway (i.e., mitophagy), which degrades the whole mitochondrion [52,53]. UPS and mitophagy share a common key element, namely ubiquitin, which covalently binds the substrates which are thus targeted for degradation and removal [54]. 

#### 2.1.1. Ubiquitin Proteasome System (UPS)

UPS promotes ubiquitination, a multistep and ATP-dependent mechanism, through the activity of three enzymes: E1, which activates ubiquitin; E2, which conjugates ubiquitin; and E3, ubiquitin ligases. A polyubiquitin chain, created by successive ubiquitination reactions, is then able to interact with the proteasome leading the substrate degradation [55]. Deubiquitinating enzymes ensure the reversibility of the entire process [56,57]. UPS dynamically regulates the mitochondrial proteome, which depends on both the importation of newly synthesized proteins from the cytosol and their degradation. Indeed, this quality control system extracts ubiquitinated proteins from the OMM and/or IMM, and degrades non-imported mitochondrial proteins [58]. In the specific case of cytosolic UPS, it controls the delivery of functional proteins to the mitochondria. Accordingly, cardiac diseases that involve the perturbation of protein homeostasis, i.e., proteostasis, alter mitochondrial function and activate death processes [59,60]. Moreover, data obtained from animal models and from human patients demonstrates that a proteasomal inefficiency, together with increased levels of protein ubiquitination, correlates with cardiomyopathies [61,62]. Accessible proteins of the OMM may be degraded by UPS, after ubiquitination, extraction from the OMM, and delivery to the proteasome, producing significative effects not only on mitochondrial morphology but also on apoptosis. For instance, when UPS induces the degradation of MCL-1, an anti-apoptotic molecule, the apoptotic proteins BAX/BAK are activated [63]. The turnover of mitochondrial proteins is also guaranteed by the translocase of the OMM, involved in the exportation of proteins localized in the intermembrane space [11,64]. Furthermore, UPS also controls nuclear-encoded mitochondrial proteins before their transport into the organelle by TOM/TIM complexes [64]. Since nuclear-encoded mitochondrial proteins are transported in an unfolded state, mitochondria possess an intrinsic quality control system, composed of chaperones and proteases, able to avoid the accumulation of misfolded or damaged proteins [65,66]. When these quality control systems fail to compensate for the excessive generation/accumulation of misfolded proteins, the mitochondrial unfolded protein response (URPmt) is activated. URPmt activates a nuclear transcriptional program that aims to restore mitochondrial homeostasis, inducing both proteases and chaperones [67]. 

#### 2.1.2. Mitophagy

When the total protein injury overcomes the restorative ability of the URPmt and UPS quality control systems, mitochondria are driven to mitophagy. The importance of mitophagy as a crucial cardiac mitochondrial quality control mechanism has been widely reported [68]. In general, autophagy represents the main degradation mechanism in cells and uses autophagosome vesicles to deliver cytoplasmic elements to the lysosomes. In this context, mitophagy is a fine-tuned process that supports the previously mentioned mitochondrial quality control systems, selectively removing damaged mitochondria. Compared to non-selective autophagy, mitophagy shows a complex organization that relies on two main events: (i) identification and labeling of mitochondria that have to be degraded; and (ii) generation of vesicular structures that transport mitochondria to lysosomes [69]. The leading processes that drive mitophagy are the PTEN-induced putative kinase 1 (PINK1)/Parkin pathway and the OMM mitophagy receptors. Especially in the heart, where inefficient mitochondria need to be degraded in order to prevent cardiomyocyte death and cardiac diseases, the PINK1/Parkin-dependent mitophagy plays a pivotal role. For instance, in the hearts of mice that were fed a high-fat diet, mitophagy increased and Parkin deficiency worsened diabetic cardiomyopathy [70]. Additionally, the PINK1/Parkin pathway is stimulated by cardiac pressure overload [71,72], during I/R [73], and under myocardial infarction [74]. The Parkin gene encodes an E3 ubiquitin ligase that interacts with E2 ubiquitin, the enzyme promoting the ubiquitination and the final removal and degradation of targeted proteins [68,75]. Mitofusin (MFN2), which will be discussed later, seems to be necessary for this mitochondrial quality control process, and is supposed to act as a mitochondrial receptor for Parkin [76]. Gong et al. elegantly demonstrated that when PINK1, located on the mitochondria, phosphorylates MFN2, it recruits cytosolic Parkin, which, in turn, ubiquitinates outer membrane proteins which are then able to interact, via protein p62, with the autophagosomal LC-3 [77]. Notably, LC-3, i.e., the microtubule-associated protein 1 light chain, has been identified by Kabeya et al. as the first mammalian protein associated with the membranes of autophagosomes [78]. A few years later, LC-3 was characterized as a crucial protein involved in the binding of PINK1 during mitophagy [79].

PINK1 promotes Parkin translocation into the mitochondria by its phosphorylation, a fundamental step for its recruitment and for the resulting ubiquitination of additional proteins, such as mitofusin 2 (MFN2), which will be discussed later [75,76,77]. Ubiquitination represents the key signal for the binding of mitophagy proteins such as sequestosome 1 (p62/SQSTM1), a so-called autophagy adaptor, providing a molecular link able to concurrently bind ubiquitin and specific proteins located on the autophagosome [80]. Autophagy adaptor proteins are characterized by a ubiquitin binding domain (UBD) and by the presence of an LC-3-interacting region (LIR), both needed to address mitochondria to their autophagosome sequestration and subsequent elimination through the lysosome intervention [53,81]. 

### 2.2. Mitochondrial Dynamics

Despite the fact that mitochondria were previously considered independent, static, and isolated organelles, it is now accepted that they form a dynamic network inside the cell, maintained by “mitochondrial dynamics”. Mitochondrial dynamics refers to the ability of mitochondria to undergo continuous cycles of fusion, during which segregated mitochondria join; and fission, during which the mitochondria divide [82]. Accordingly, mitochondria are highly dynamic organelles, whose function is dynamically regulated by their fusion and fission, movement along the cytoskeleton, and mitophagy. These processes are essential to maintaining normal mitochondrial morphology, distribution, and function—including mitochondrial respiration, mitochondrial metabolism, and ROS production—as well as normal cell metabolism [83].

Selective mitochondrial fusion proteins known as membrane-anchored dynamin family members, which are abundantly expressed in the adult heart, mediate the fusion of two adjacent mitochondria to form a more elongated mitochondrion; in particular, fusion is promoted by mitofusin-1 (MFN1) and MFN2 proteins, whose normal functions rely on the activity of guanosine triphosphatases (GTPases), by forming stable homo-oligomeric and hetero-oligomeric complexes through their GTPase domain at the outer mitochondrial membrane, and optic atrophy 1 (OPA1), which is located in the IMM and in the intermembrane space; OPA1 is a dynamin-like GTPase that is anchored to the IMM by an N-terminal transmembrane domain, and mediates IMM fusion, enhancing the interconnection of the mitochondrial network [84,85]. Mitochondrial fusion allows the exchange of intramitochondrial material (i.e., mitochondrial DNA (mtDNA), proteins, lipids, and metabolites), necessary for maintaining a balanced pool of mitochondrial protein, as well as a genetic and biochemical homogeneity within the mitochondrial population [83].

On the other hand, mitochondrial fission proteins participate in mitochondrial fission, a multistep and complex process that divides a single mitochondrion into two mitochondria; the key factor mediating mitochondrial fission is dynamin-related protein 1 (Drp1), a homologous protein of GTPase power protein, which is recruited from the cytosol to the OMM by various OMM-anchored adapter proteins, including fission protein 1 (Fis1) and mitochondrial fission factor (MFF), which act as Drp1 receptors [8,86]. Mitochondrial fission is necessary to replicate the mitochondria during cell division, to facilitate the transport and distribution of mitochondria, and to permit the isolation of damaged mitochondria for mitophagy. Alterations of mitochondrial dynamics lead to cardiac mitochondrial integrity and mtDNA damage, and cell death ultimately occurs [87].

In the case of prolonged exposure of the heart to stressful conditions, such as hypoxia, ischemia/reperfusion, oxidative and nitrosative stress, and hyperglycemia, the profound alterations of mitochondrial dynamics and mitophagy lead to irreversible damage of the mtDNA and excessive ROS released by damaged mitochondria, ultimately leading to cardiotoxicity [87,88]. 

## 3. Cardiac Mitochondrial Dysfunction Secondary to Anti-Cancer Drug Treatments

It is well-established that the cardiotoxic side effects of several anti-cancer therapies are frequently mediated by mitochondrial damage [89]. This evidence was first demonstrated through the detrimental effects of chemotherapy on skeletal muscle, a tissue in which the number of mitochondria is very high, although lower than cardiomyocytes [90]. Accordingly, skeletal muscle weakness, together with persistent fatigue, are common in cancer patients undergoing chemotherapy, and some of the skeletal-muscle-specific symptoms are due to mitochondrial dysfunction [12,91,92]. At the molecular level, different processes, including but not limited to oxidative stress, inflammation, immunometabolism, pyroptosis, and autophagy, act together, promoting chemotherapy-induced multifactorial cardiotoxicity [93].

In this context, growing evidence highlights the involvement of diverse mechanisms that mainly converge on mitochondrial dysfunction. There are a number of potential reasons why cardiac mitochondria represent a major target of antineoplastic drugs. Firstly, cardiomyocytes show a high susceptibility to oxidative stress because they are rich in mitochondria and possess relatively low endogenous antioxidant defense systems [94]; additionally, they use enormous amounts of ATP, whose production occurs in mitochondria and is maintained, as discussed above, by mitochondrial biogenesis, replication, and autophagy/mitophagy [95]. Overall, mechanisms that induce mitochondrial toxicity via anti-tumor agents are many, and mostly related to the alterations occurring in ROS/redox system regulation, the mitochondrial calcium homeostasis system, mitochondrial dynamics, and endoplasmic reticulum (ER) stress signaling, all processes linked by a vicious cycle that disrupts cardiac cell homeostasis and induces cell death [96,97]. 

In the following paragraphs, we will analyze the main mitochondrial determinants of cardiotoxicity secondary to three major classes of antineoplastic drugs widely reported as cardiotoxic, represented by ANTs, RTKIs and PIs.

### 3.1. Anthracyclines (ANTs)

**Figure 1:**
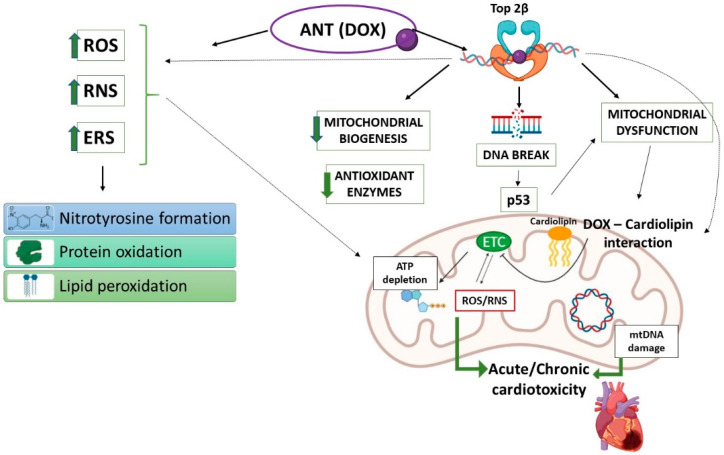
Schematic representation of major events leading to mitochondrial dysfunction during ANT (DOX)-induced cardiotoxicity. ANT: anthracycline; DOX: doxorubicin; ROS: reactive oxygen species; RNS: reactive nitrogen species; ERS: endoplasmic reticulum stress; Top2β: topoisomerase 2β; ETC: electron transport chain; mtDNA: mitochondrial DNA.

ANTs, primarily doxorubicin (DOX), are antibiotics that exert their anti-tumor activity by inducing single- and double-strand breaks in DNA, preventing DNA synthesis, intercalating with DNA base pairs and stabilizing the topoisomerase (Top) 2α complex after DNA cleavage [98,99]. ANTs still represent the cornerstone of treatment in many malignancies, including lymphomas, leukemias, sarcomas, advanced and early breast cancer, and small cell lung cancer [100,101]. However, the clinical use of ANTs is seriously hampered by dose-related cardiomyocyte injury and death, leading to left ventricular dysfunction and heart failure, representing the most clinically-limiting adverse feature of ANTs [94,100,101,102]. The most relevant ANT-related cardiac dysfunction from a cardio-oncological point of view involves the myocardium, and is manifested by a decreased left-ventricular ejection fraction, which may progress to congestive heart failure [103]. Mechanically, cardiac dysfunction induced by ANTs relies on alteration in iron metabolism and ROS, and reactive nitrogen species (RNS) overproduction; however, intriguing evidence emerged in recent years indicating that ANTs may use alternative damaging mechanisms, such as Top 2β inhibition, inflammation, immunometabolism, pyroptosis, and autophagy, which explains, at least in part, the complexity of iatrogenic ANT-induced progressive cardiomyopathy and heart failure (Figure 1) [104]. On the other hand, ANTs typically associate with an irreversible form of cardiac dysfunction (known as type I cardiotoxicity) characterized by evident ultrastructural myocardial abnormalities, as evinced by vacuoles, myofibrillar disarray and dropout, and myocyte necrosis at higher cumulative doses [103]. 

Although the pathogenetic mechanisms accounting for ANT-dependent cardiotoxicity remain complex and multifactorial, mitochondrial oxidative stress, in addition to the redox cycling secondary to ANT-iron complex formation, and targeting of Top 2β (one of the two types of Top2 present in quiescent non-proliferating cells, including cardiomyocytes), are the most relevant. The inhibition of Top 2β by ANTs causes double-stranded DNA breaks and the consequent activation of the tumor suppressor protein p53, strongly contributing to the development of cardiotoxicity [93,105,106]. Importantly, over the last decades, a large number of studies reported sub-chronic/chronic mitochondrial cardiac alterations, in terms of disrupted mitochondrial calcium homeostasis [107,108] and mitochondrial respiration alteration [109,110] during DOX exposure in both pre-clinical and human models. The primary effect of DOX on mitochondrial activity is related to its capacity to interfere with oxidative phosphorylation and inhibit ATP synthesis. In particular, DOX can inhibit mitochondrial Complex I by diverting electrons from NADH to molecular oxygen, leading to DOX recycling and generating a futile cycle, a major ROS production site in DOX-induced toxicity [111,112]. Other evidence subsequently demonstrated that DOX also interferes with Complexes III and IV, the phosphate carrier and the adenine nucleotide translocator [112]. Free radicals derived from DOX redox cycling are responsible for many of the secondary effects of oxidative stress induced by DOX; these include alteration of macromolecules as well as depletion of GSH and pyridine nucleotide reducing equivalents [113]. The generation of excessive ROS and RNS overcomes the endogenous capacity in producing antioxidant enzymes, including mitochondrial antioxidant systems, leading to the typical redox modifications of macromolecules, including nitrotyrosine formation, protein carbonylation, and lipid peroxidation (Figure 1) [93,102,114]. In addition to the lower antioxidant surplus in the heart respective to other tissues, the ability of DOX to accumulate primarily in mitochondria and nuclei [115] can explain the cardio-selective toxicity of the drug. In this context, it should also be noted that ANTs are able to selectively bind the phospholipid cardiolipin, localized in the IMM, in close proximity to the mitochondrial electron-transport chain, leading to mitochondrial accumulation of the drug (Figure 1) [116]. Cardiolipin is an acidic phospholipid that plays a crucial role in the regulation of mitochondrial function, structure, and dynamics, and mitochondrial dysfunction in different CVDs correlate with cardiolipin remodeling; in particular, cardiolipin peroxidation induces mitochondrial impairments and CVD progression [117]. In this regard, several studies on animal models demonstrated that the ANT-cardiolipin interaction alters cardiolipin function since, in this condition, cardiolipin is not able to anchor cytochrome c or lipid-protein interfaces for the other important mitochondrial proteins [118]; the oxidized cardiolipin can disrupt the electron transport chain, stimulating additional ROS/RNS production and inducing mitochondrial DNA damage (Figure 1) [112]. 

As elegantly reviewed by Wallace et al., mechanistic studies showed that the acute inhibition of mitochondrial oxidative phosphorylation induced by DOX may induce compensatory selective cardiomyocyte adaptations [119]. For instance, as indicated in an acute in vitro model (i.e., H9c2 rat cardiac myoblasts), a major cellular defense mechanism secondary to DOX exposure concerns the activation of the Keap1 (kelch-like ECH-associated protein 1)/Nrf2 (Nfe2l2, nuclear factor erythroid derived 2 like 2)-antioxidant response element (ARE) signaling pathway [120]. Other in vitro reports suggest that acute DOX exposure can induce, in cardiomyocytes, the nuclear up-regulation of p66Shc, an adaptor protein modulating cellular redox status and serving as an oxidative stress sensor, in order to modulate FoxO (Forkhead box subgroup O) nuclear transcription factors, inducing cell death in order to eliminate damaged cells [121]. 

Both experimental and clinical evidence supports the hypothesis that specific antioxidants may be effective in protecting the heart from ANT toxicity, in terms of HF prevention or cardiac damage mitigation. Clinical trials and meta-analytical studies have been conducted to determine the protective effect of specific antioxidants, such as carvedilol, L-carnitine, and dexrazoxane in ANT-induced cardiomyopathy [122,123,124,125,126,127]. However, it is still unclear whether these antioxidants exert cardioprotective effects in humans without impairing the anticancer activity of ANTs; moreover, most of these studies evaluated the effects of ANTs alone, not in combination with other therapies. Therefore, larger multi-center trials are required to effectively evaluate the beneficial activity of antioxidant agents in co-administration with ANTs and other anticancer drugs [128,129].

Notably, mitochondrial alteration secondary to ANTs is profoundly interconnected with Top 2β targeting and ROS/RNS generation, since indirect effects on mitochondrial function can also occur through nuclear-mediated effects related to the inhibition of Top 2β in cardiomyocytes. Accordingly, after DNA breaks secondary to DOX-Top 2β binding, p53 stimulation also induces defective mitochondria biogenesis and metabolic impairment by decreasing the transcription of crucial genes involved in mitochondrial biogenesis and function, such as peroxisome proliferator-activated receptor gamma coactivator 1-α (PGC-1α), which is also a key regulator of SOD, and peroxisome proliferator-activated receptor gamma coactivator 1-β (PGC-1β), and alteration of oxidative phosphorylation [105]. DOX is also able to downregulate uncoupling protein 2 (UCP-2) and uncoupling protein 3 (UCP-3), members of the superfamily of mitochondrial transport proteins which regulate mitochondrial ROS production, predisposing the failing heart to oxidative stress [130]. These data are of particular interest since it has been reported that polymorphisms in the human UCP genes can affect the expression/function of the protein [131]; thus, genetic variations in human UCP-2 and/or UCP-3 may affect the susceptibility of patients to DOX-related cardiotoxicity. 

Human studies and pre-clinical models indicate that the redox and metabolic alterations, as well as mitochondrial impairment secondary to a DOX regimen, persist after therapy completion (one to five weeks following the last of six drug treatments) and that the toxic effects of DOX can propagate to successive generations of mitochondria, leading to cumulative dose-dependent and progressive mitochondrial dysfunction [132,133]. This can correlate with DOX cardiotoxicity memory, according to which myocardial mass reduction following DOX administration may predispose the heart to further alterations after subsequent DOX treatments [119,134] (Figure 1).

**Figure 2:**
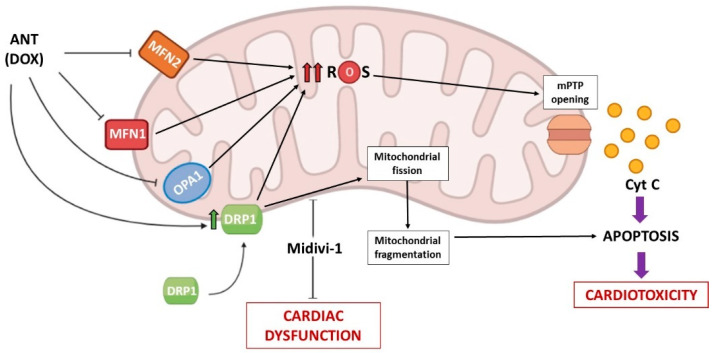
Schematic representation of mitochondrial dynamics alterations induced by ANT (DOX) leading to cardiotoxicity. ANT: anthracycline; DOX: doxorubicin; ROS: reactive oxygen species; MFN1: mitofusin-1; MFN2: mitofusin-2; OPA1: optic atrophy 1; DRP1: dynamin-related protein 1; Midivi-1: mitochondrial division inhibitor-1; mPTP: mitochondrial permeability transition pore; cyt c: cytochrome c.

There is also growing evidence that ANTs can disrupt mitochondrial dynamics, which is increasingly recognized as a major process driving ANT-dependent heart dysfunction, so that several therapeutic interventions targeting mitochondrial dynamics have shown promising effects in attenuating DOX cardiac toxicity in both cell and animal models (Figure 2). 

In vitro evidence on cultured neonatal rat cardiomyocytes demonstrated that DOX negatively affects levels of MFN2, thus promoting mitochondrial fission and ROS production, while increasing MFN2 levels counteracted these processes [135]. Similarly, other studies indicate that MFN1 and OPA1 are downregulated in response to apoptotic stimulation following DOX exposure in cardiomyocytes [136]. Conversely, DOX can upregulate the expression of mitochondrial fission protein 1 in HL-1 cardiac myocytes, while its lessening reduces DOX-dependent apoptosis, preventing dynamin 1-like accumulation in mitochondria [137]. In vivo, sub-chronic DOX treatment in rats increased mitochondrial permeability transition pore (mPTP) susceptibility and induced apoptosis, decreasing the expression of MFN1, MFN2, and OPA1, and increasing Drp1, activating autophagy and mitophagy signaling [138]. Moreover, Xia et al. (2017) demonstrated in H9c2 cardiomyocytes, as well as in a mouse model of DOX-induced cardiomyopathy, that DOX exposure augmented Drp1 and its Ser 616 phosphorylation [139]. These findings were corroborated by the ability of both LCZ696, a novel angiotensin receptor-neprilysin inhibitor, and of mitochondrial division inhibitor-1 (Midivi-1), a specific inhibitor of Drp1, to mitigate the DOX-dependent mitochondrial dynamics alterations and cardiac dysfunction (Figure 2). On the other hand, the overexpression of Drp1 antagonized the beneficial effect of LCZ696 in vitro [139]. The crucial involvement of Drp1 in DOX-dependent cardiotoxicity was further demonstrated by Zhuang et al. (2021) [140], who confirmed that the expression of Drp1 increased following DOX treatment both in vitro and in vivo, leading to apoptosis of cardiomyocytes. In this study, the authors also found that an overexpression of Klotho (an anti-aging protein whose defects in its gene expression accelerated cardiac hypertrophy and remodeling in mice and human vascular calcification) [141,142] or Midivi-1 can trigger cardioprotection through inhibition of cell death and reversal of mitochondrial dynamics perturbation.

Consistently, other in vitro and in vivo reports strongly support a key role for Drp1-dependent mitochondrial fragmentation in DOX-dependent cardiomyopathy. Catanzaro et al. (2019) indicated that a short interference-RNA-mediated knockdown of Drp1 prevents DOX-induced mitochondrial fragmentation, mitophagy flux, and apoptosis in H9c2 cells, while Drp1-deficient mice were protected from DOX-induced cardiac dysfunction [143]. Various studies reported that Drp1 can be reversibly phosphorylated at its serine residues, and that this phosphorylation strongly affects both the localization and activation of cardiac Drp1 [144]. Specifically, when Drp1 is phosphorylated at Ser 637, its translocation to mitochondria is prevented and mitochondrial fission is inhibited [145]. In this regard, a very recent study identified the cardiomyocyte mitochondrial dynamic-related lncRNA 1 (CMDL-1) as the most significantly downregulated long non-coding RNA (lncRNA) in cardiomyocytes after DOX exposure, and demonstrated that CMDL-1 can inhibit Drp1 translocation to mitochondria by promoting Drp1 Ser 637 phosphorylation, thereby preventing mitochondrial fission and apoptosis [146]. 

Among the different OMM proteins that promote mitochondrial fission by recruiting Drp1 to the mitochondrial surface, it has also been shown that mitochondrial dynamics proteins of 49 kDa (MiD49, MIEF2) can participate in the regulation of cardiac mitochondrial dynamics during DOX treatment. Accordingly, recent studies identified MIEF2 as a transcriptional target of the transcription factor FoxO3a, and reported that FoxO3a can prevent DOX-induced mitochondrial fission, apoptosis, and cardiotoxicity by suppressing MIEF2 expression [147].

Overall, these data indicate that DOX displays inhibitory effects on mitochondrial fusion while promoting mitochondrial fission; in particular, the increased Drp1 expression, whose protein levels were previously found increased in patients with ischemic cardiomyopathy and dilated cardiomyopathy [148], represents a key factor also promoting the shift toward mitochondrial fission during DOX exposure.

Taken together, these observations suggest that preventing mitochondrial fission and targeting mitochondrial dynamics could represent a promising strategy in saving cardiomyocyte loss due to DOX-induced cardiotoxicity (Figure 2).

### 3.2. RTK Inhibitors (RTKIs)

Receptor tyrosine kinases (RTKs) are cell surface transmembrane proteins activated in response to ligand binding, an event conveying downstream stimulatory signals towards cell proliferation, migration, invasion, differentiation, and angiogenesis [149]. Aberrant RTK signaling, which may occur in response to genome amplification, gain of function mutations, or chromosome rearrangements, has been shown to contribute to tumor development and progression, as well as to anti-cancer treatment failure [149,150]. Most of the known human RTKs share a similar protein structure, with an extracellular ligand-binding (N)-terminal domain, a single spanning transmembrane helix, and an intracellular carboxyl(C)-terminal domain [151,152]. A number of pharmacological approaches have been proposed to block aberrant RTK signaling in cancer, including the use of monoclonal antibodies targeting either specific receptors or their ligands, as well as the use of RTKIs’ small molecules.

RTKIs mainly act by preventing receptor autophosphorylation through interference with the ATP binding site within the kinase catalytic domain of the protein; nevertheless, certain RTKIs are non-ATP competitors [153]. One of the clinical advantages of targeting aberrant RTK signaling is that fewer off-target effects are to be expected when using targeted therapies compared with chemo- and radiotherapy. Despite the risk of developing cardiovascular effects appearing to be generally low, long-term use of certain RTKIs can significantly increase the risk of cardiovascular events. Such effects appear to be highly variable among the class of RTKIs, although it is generally accepted that pre-existing cardiac pathological conditions, such as hypertension, hyperlipidemia, and diabetes, as well as both the genetic background and immune status of the patient, may influence the risk and severity of RTKI-associated cardiovascular toxicity [154].

RTKI-triggered cardiovascular side effects range from asymptomatic left ventricular dysfunction to symptomatic congestive heart failure, arrhythmia/QT prolongation, hypertension, and acute coronary syndrome [155]. Despite the fact that the mechanisms are various and drug-specific side effects are observed, a general model of toxicity involves both on-target and off-target effects.

The most important pharmacological strategy aimed at blocking tumor angiogenesis is the targeting of the vascular endothelial growth factor (VEGF)/VEGFR transduction pathway. Both anti-VEGF monoclonal antibodies and VEGFR small molecule inhibitors have been shown to induce left ventricular dysfunction, ischemia, and thromboembolic events [156]. Commonly, the most strongly observed effect in response to anti-VEGF therapies is hypertension, which is due to unbalanced production in blood pressure regulators (i.e., increased endothelin-1 and decreased nitric oxide production, respectively), as well as reduced capillary density [157]. It is worth mentioning that certain detrimental cardiovascular effects induced by RTKIs are directly attributable to loss of RTK function and therefore compromised cardiomyocyte biology. This is the case for anticancer therapies that target the ERBB family of RTKs [158].

As ERBB family members play a crucial role in the maintenance of cardiomyocytes’ homeostasis and cell response to stress and injury, the disruption of their transduction network results in myocyte dysfunction. For instance, interfering with ERBB-mediated signaling may promote the mitochondrial release of cytochrome c [159], together with the inhibition of antiapoptotic pathways, the induction of caspase activation, and the subsequent activating of apoptotic cell death [160]. Additional studies have shown that the monoclonal antibody trastuzumab, which targets ERBB family members, may compromise the ability of cardiomyocytes to cope with stress, including pressure overload and/or ANT injury, thus providing a rationale for the increased risk of cardiotoxicity of the drug combination (trastuzumab plus ANT) compared to single agent treatment [161].

**Figure 3:**
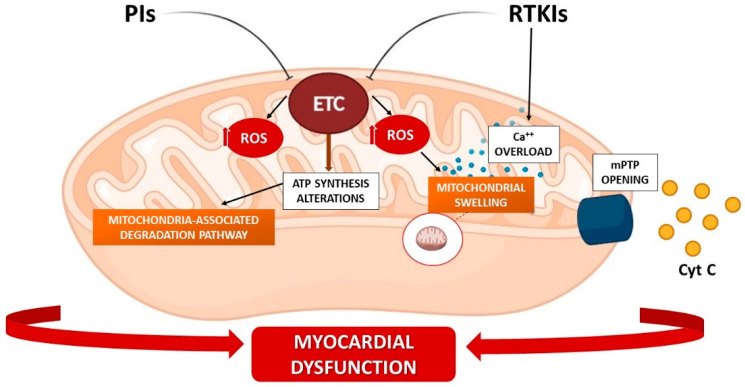
Proposed mechanism of cardiac mitochondrial alterations secondary to PIs and RTKIs exposure. PIs: Proteasome inhibitors; RTKIs: Receptor tyrosine kinase inhibitors; ETC: electron transport chain; ROS: reactive oxygen species; mPTP: mitochondrial permeability transition pore; cyt c: cytochrome c.

Interestingly, cardiac toxicity has also been detected after inhibition of non-receptor TKs. For instance, imatinib mesylate, which mainly targets the fusion protein bcr-Abl and represents the drug of choice in chronic myelogenous leukemia (CML) and Philadelphia chromosome-positive B-acute lymphoblastic leukemia (Ph+ B-ALL), induces myocyte dysfunctions resulting in severe CHF [162]. The analysis of endomyocardial bioptic tissue obtained from patients who developed CHF after treatment with imatinib mesylate revealed profound ultrastructural mitochondrial changes and abnormalities, including pleomorphisms, swelling, and erosions of cristae, together with intense cytosolic signs of cell stress, like formation of vacuoles [162]. Cardiomyocytes cultured with imatinib mesylate had high ER stress, deep alterations of mitochondrial membrane potential, reduction of ATP production, release of cytochrome c into the cytosol, and activation of cell death programs (Figure 3) [157,162]. Of note, myocytes’ mitochondrial damage and subsequent energy rundown may also be attributable to the impaired activity of the energy-restoring AMP-activated protein kinase (AMPK), a frequently observed off-target effect of RTKIs [163].

Further corroborating these findings, deranged mitochondrial energetics were also observed in response to clinically relevant concentrations of sorafenib, which compromised oxidative phosphorylation by inhibiting complexes V, II, and III of the electron transport chain [164,165], thereby halting ATP production necessary for myocyte contractility (Figure 3).

Of note, promising clinical effects of the multi-targeting TKI ponatinib, approved for the treatment of CML and Ph+ B-ALL [166], have been mitigated by the cardiac-specific toxicity induced by this drug, including myocardial infarction, severe congestive heart failure, and cardiac arrhythmias. 

A well-designed approach by Talbert et al. demonstrated that the cardiac toxicity potential of ponatinib is reflected by dramatic changes in ROS generation and lipid formation, consistent with mitochondrial impairment and metabolic imbalances [167]. In addition, the authors developed a comprehensive in vitro screening tool based on the use of human-induced pluripotent stem cell-derived cardiomyocytes (hiPSC-CM), which was able to accurately predict human cardiac toxicity by evaluating several indices, including signs of mitochondrial stress [167].

Likewise, enhanced ROS generation and oxidative stress are largely implicated in the initiation of mitochondrial dysfunction, which triggers cell damage in a broad range of cellular components. It should be mentioned that certain RTKIs promote mitochondrial dysfunctions in an indirect fashion. This is the case for regorafenib, a drug approved for metastatic colorectal cancer and advanced gastrointestinal stromal tumors, which disrupts calcium homeostasis, thereby inducing mitochondrial swelling due to calcium overload [168].

On the other hand, abnormalities in mitochondrial structures and function may result as a consequence of RTKIs’ action on several off-target kinases, including c-Jun N-terminal kinase, protein kinase A and pyruvate dehydrogenase kinase (PDK); moreover, PDK, a mitochondrial enzyme acting with pyruvate dehydrogenase phosphatase to regulate pyruvate dehydrogenase complex, has been shown a promising therapeutic target in complex diseases including diabetes, heart failure, and cancer, as well as in the mitochondrial toxicity induced by RTKIs [169,170]. Accordingly, the inhibition of these signaling pathways may disrupt oxidative phosphorylation, and facilitate the establishment of both morphological abnormalities consistent with hypertrophic responses and the shift of energetic metabolism toward anaerobic dependency [171].

Clearly, the disruption of mitochondrial structure and function represents the main trigger for cardiomyocytes’ metabolic reprogramming, as nicely shown by Wang et al., who performed a systems-level analysis of human cardiomyocytes differentiated from hiPSCs and exposed to different RTKIs [172]. Results showed a parallel inhibition of mitochondrial ATP production and an increase in glycolysis after treatment with RTKIs [172]. The effect on mitochondrial functionality appeared to be reversible upon drug withdrawal, and the metabolic remodeling toward the glycolytic pathway served as an alternate route to cope with metabolic stress. Likewise, an increased tendency to rely on glycolysis is a peculiar feature of hypertrophic myocardium and myocardial ischemia, as well as heart failure [173]. Despite the fact that the mechanisms involved in RTKI cardiotoxicity are an active topic under investigation, and less-known than other anti-cancer drugs like ANTs, a relative lack of adequate pre-clinical platforms to predict, detect and hamper drug-associated cardiovascular effects still represents a challenge to basic researchers and clinicians in this field. Therefore, additional effort has to be implemented to minimize the detrimental cardiac effects of RTK inhibition, taking into account the complexity of the RTK signaling networks. For instance, the inhibition of EGFR by gefitinib (mainly used for the treatment of non-small cell lung cancer), has been shown to induce mitochondrial membrane potential alteration, cellular plasma membrane permeabilization, and activation of apoptosis in cardiomyocytes [174]. These effects are triggered by the CYP1A1-dependent formation of toxic reactive metabolites within myocytes’ microsomes. It is worth recalling that in various contexts, EGFR cooperates with other non-RTK transduction partners to promote biological responses. This is the case for the G-protein coupled receptor 30, namely GPER, which serves as an alternate receptor for estrogens [175,176]. Numerous studies have demonstrated that GPER activation elicits beneficial cardiovascular effects by regulating myocyte cell response to stressful conditions, including ischemia, inflammation, and hypertension [177,178]. Additionally, GPER activation has been shown to reduce DOX cardiotoxicity [179]. Table 1 summarizes the main RTKIs and their cardiovascular toxicity.

### 3.3. Proteasome Inhibitors (PIs)

As mentioned above, UPS, a crucial mechanism for protein degradation, regulates protein turnover, thus affecting various cellular functions [190]. UPS is a relevant therapeutic target in cancer, especially in hematological malignancies like multiple myeloma (MM), a cancer of terminally differentiated plasma cells accumulating in the bone marrow [191,192]. Since plasma cells produce high amounts of immunoglobulins, they are very sensitive to the deregulation of protein degradation; moreover, malignant plasma cells appear even more susceptible to proteasomal inhibition than the healthy ones, due to constitutive activation of the oncogenic NF-κB pathway [193]. In fact, PIs act by blocking IκB degradation and thus, indirectly, inhibiting NF-κB signaling, although other processes are emerging, which contribute to the antitumor effects of PIs, and include inhibition of altered cell cycle control and apoptosis, ER stress, angiogenesis, and DNA repair [194], as well as epigenetic modulating effects [195,196].

The striking sensitivity of malignant cells to PIs has led to their approval for MM treatment, with three drugs being routinely used in a clinical setting [197] in association with other anti-MM therapies such as dexamethasone, and immunomodulatory drugs (lenalidomide), chemotherapy (DOX, mephalan, or cyclophosphamide), antibodies (elotuzumab or daratumumab), or histone deacetylase inhibitors (panobinostat) [198]. The first-in-class PI was bortezomib, a boronic acid derivative acting as a slowly reversible inhibitor of the β5 catalytic proteasomal subunit. Next, the irreversible inhibitor of β5 site carfilzomib and the first oral PI, ixazomib, were approved [197]. 

Although the toxicity of PIs is well-controlled in a clinical setting, distinct adverse profiles (such as peripheral neuropathy and cardiotoxicity) frequently arise and can lead to early discontinuation of the therapy [199]. 

The cardiotoxicity of bortezomib is still under debate, and likely depends on whether the drug is administered in patients with significant cardiovascular disease risk factors or previously treated with known cardiotoxic chemotherapeutics [200]. 

Molecular mechanisms involved in bortezomib-induced cardiovascular toxicity remain to be fully elucidated. In rat cardiomyoblast H9c2 cells, bortezomib causes the accumulation of polyubiquitinated proteins which, in turn, leads to ER stress and compensatory autophagy [201]. MG262, another boronic acid-based PI, promotes the translocation of the nuclear factor of activated T-cells (NFAT) in neonatal rat ventricular myocytes through the activation of the calcineurin-NFAT pathway, with significant changes in the cell morphology [202]; moreover, the inhibition of the proteasome by bortezomib in primary neonatal rat ventricular myocytes activates caspase-3 and caspase-7, triggering apoptosis [203]. Notably, mitochondria have been identified as a relevant target of cardiotoxicity because bortezomib inhibits complex V of the respiratory chain, resulting in a drop in ATP synthesis in the hearts of treated rats, and in a decreased cell shortening of primary rat left ventricular myocytes [204]. Functional and reversible changes accompanied the structural alterations of the mitochondria, which become pleomorphic and enlarged with concentric cristae and electron-dense inclusions, and showing misalignment of the myofibrillar network [201]. Moreover, bortezomib-mediated mitochondrial dysfunction might also be explained by the recently described process of extraction of misfolded proteins from mitochondria, and their subsequent degradation in proteasomes, called mitochondria-associated degradation (MAD) [205]; inhibition of proteasome leads to accumulation of misfolded and damaged proteins in mitochondria, resulting in their dysfunction (Figure 3).

The cardiovascular effects of bortezomib have been also addressed in several in vivo preclinical models that led to contradictory results. In fact, left ventricular systolic and diastolic function was preserved and no morphological myocardial abnormalities were detectable in adult male rabbits upon exposure of bortezomib [206]; conversely, male Wistar rats treated with bortezomib developed a reversible cardiac dysfunction with a significant decrease in left ventricular ejection fraction [201]. 

In cancer patients, the cardiovascular AEs associated with bortezomib treatment so far include heart failure, conduction disorders such as complete atrioventricular block, arrhythmias including atrial fibrillation, ischemic heart disease, pericardial effusion, and orthostatic hypotension [207]. A systematic review and meta-analysis of 25 prospective phase II/III trials evaluating bortezomib in different malignancies indicated that it does not significantly increase the risk of cardiac AEs as compared to control medications [208]. The overall cardiac safety profile of bortezomib was confirmed in a later retrospective analysis of patients included in the phase II registration study for US and EU regulatory approval, and in all phase III studies that led to US and EU approval of the drug [209], reporting no significant differences in the incidence of cardiovascular toxicities between bortezomib- and non-bortezomib-based arms [207].

Carfilzomib, which binds irreversibly to β5 (chymotryptic-like activity) and β5i immunoproteasome, was found to have greater selectivity for β5 subunits, with minimal affinity to β1 and β2 subunits when compared with bortezomib [210]. Carfilzomib induced proteasome inhibition in excess of 80% of patients [211], and its efficacy in bortezomib-resistant cells was likely due to prolonged and sustained inhibition of the proteasome. Carfilzomib received FDA approval in 2012 for use in relapsed and refractory MM (RRMM) patients who had previously received at least two therapies. Overall, several studies of carfilzomib noted an increased risk of cardiovascular AEs. A pooled analysis of phase II studies with carfilzomib showed 22% of patients developing cardiac side effects, such as arrhythmias, heart failure, treatment-associated cardiomyopathy, and ischemic heart disease [212]. In 2015, a carfilzomib combination regimen with lenalidomide and dexamethasone (KRd) was approved by the FDA for RRMM with one or more prior lines of treatment, based on significantly improved PFS and improved quality of life in a phase III trial [213,214]. However, this trial (ASPIRE) reported that the combination with the immunomodulatory drug lenalidomide increased cases of CVAEs, such as hypertension rates, heart failure rate, and ischemic heart disease rates [212,215]. The higher potency and irreversible inhibition by carfilzomib, along with dose-limiting neuropathy associated with bortezomib, may be the link between carfilzomib and higher incidences of CVAEs. In a systemic review and meta-analysis of 24 prospective clinical trials that included 2594 patients, a large range of reported CVAEs, with all grades of CVAE ranging from 0 to 52% and high-grade CVAEs ranging from 0 to 45% [216] was found. In an effort to better define risk factors and outcomes in patients who receive PI therapy, a prospective, observational study (PROTECT), was conducted [217], in which patients underwent baseline assessments over 6 months of bortezomib or carfilzomib; cardiac biomarkers included troponin I or T, BNP, NT-proBNP, ECG, and echocardiography. Of the CVAEs, 51% were in patients treated with carfilzomib, and 17% of those were treated with bortezomib, confirming the superior cardiotoxicity profile of carfilzomib. The study also demonstrated an association between BNP and NT-proBNP rise and increased CVAE risk. Overall, this trial reported a much higher incidence of CVAEs than prior studies, possibly due to its prospective nature as well as to the fact that CVAEs were captured as primary endpoint, showing that cardiotoxicity mainly occurred in patients with cardiac comorbidities.

Ixazomib (MLN9708), like bortezomib, acts as a reversible inhibitor on the β5 (chymotrypsin-like) and β5i subunits of the immunoproteasome, with additional inhibition of β1 and β2 subunits at higher concentrations [218,219]; it was the first orally bioavailable drug approved by the FDA in 2015 for RRMM, used in combination with lenalidomide and dexamethasone for MM patients in which one or more prior lines of treatment failed. It showed a pattern of cardiovascular AEs similar to bortezomib, although the trial excluded patients with cardiac comorbidities [220,221]. 

To overcome the cardiotoxicity of PIs like carfilzomib, mitochondrial functions affected by PIs are being dissected, and novel PIs devoid of cardiotoxicity are also being developed and analyzed in preclinical studies [222]. Combination strategies reducing PI doses are currently being evaluated in clinical trials to counteract dose-dependent CVAEs [223]. 

Table 2 recapitulates the main PIs used in clinical settings and their relative cardiotoxic effects, as well as the potential preventive/cardioprotective strategies to reduce their CVAEs.

## 4. Conclusions

Cardiotoxicity associated with widely used anticancer drugs, such as ANTs, RTKIs, and PIs, still represents a significant clinical challenge that compromises the quality of life and overall survival of cancer patients. Although the mechanisms driving the cardiotoxicity of these anticancer drugs is multifactorial, and different pathways seem implicated, a growing line of evidence strongly suggests that the cardiac AEs from these anticancer therapeutics involve direct or indirect mitochondria-related toxicity. In addition to the ability of the anticancer drugs to affect mitochondrial bioenergetics, mitochondrial DNA replication, mitochondrial oxidative/nitrative stress, and cell death, emerging evidence also underscores dysregulated mitochondrial dynamics as determinant of anticancer-drug-dependent cardiotoxicity. A thorough understanding of the mitochondrial processes underlying cardiovascular toxicity is therefore fundamental to rationally develop effective strategies preventing cardiomyocyte dysfunction or loss elicited by several chemotherapeutic regimens.

## Figures and Tables

**Table 1:** List of main RTKIs and their cardiovascular toxicity.

Tyrosine Kinase Inhibitor	Molecular Target	Type of Study	Type of Cancer	Cardiotoxic Effect	Ref.
**Sunitinib**	Multi-tyrosine kinases (VEGFR, PDGFR, c-KIT)	Phase I/II clinical trial Multicenter prospective study	Imatinib-resistant, metastatic, gastrointestinal stromal tumors metastatic renal cell carcinoma	Left ventricular dysfunction congestive heart failure hypertension	[157,180]
**Pazopanib**	Multi-tyrosine kinases (VEGFR, PDGFR, c-KIT)	Randomized, double-blind, placebo-controlled study	Advanced solid tumors	Hypertension reduction in heart rate small prolongation of the QTc interval	[181]
**Sorafenib**	Multi-tyrosine kinases (VEGFR, PDGFR, FLT3)	Systematic review and meta-analysis	Renal cell carcinoma melanoma	Hypertension myocardial infarction ischemia acute coronary syndrome rarely heart failure	[182]
**Regorafenib**	Multi-tyrosine kinases (VEGFR1-3, PDGFR-β, FGFR)	Meta-analysis of 45 RTCs	Solid tumors	Hypertension generally few cardiovascular side effects	[183]
**Ponatinib**	Multi-tyrosine kinases FGFR, PDGFR, and VEGFR	Phase II clinical trial Review	Chronic myeloid leukemia; Philadelphia chromosome-positive leukemias	Arterial thrombotic events	[184,185]
**Cabozantinib**	Flt-3, RET, MET	Multicenter prospective study Review	Metastatic renal cell carcinoma medullary thyroid cancer	Modest risk of developing left ventricular systolic dysfunction hypertension	[186,187]
**Nilotinib**	PDGFR, CSF-1R,	Retrospective study	Chronic myeloid leukemia	Accelerated atherosclerosis peripheral arterial occlusive disease (PAOD) QTc prolongation.	[188]
**Axitinib**	VEGFR	Clinical trial	Metastatic renal cell carcinoma	Hypertension myocardial infarction	[189]

**Table 2:** Main PIs, associated CVAEs, and potential preventive/cardioprotective strategies to reduce cardiotoxicity.

Proteasome Inhibitors	Mechanism of Action	Type of Study	Type of Cancer	Cardiotoxic Effects	Potential Preventive/Cardioprotective Strategies	Ref.
**Bortezomib**	Slowly-reversible inhibitor of β5 and β5i subunits	Systematic review and meta-analysis of 25 prospective phase II/III trials	Untreated multiple myeloma	Heart failure, conduction disorders, arrhythmias, ischemic heart disease, pericardial effusion and orthostatic hypotension	Assessment of cardiac function, evaluation of serum biomarkers of heart failure; Evaluation of atrial fibrillation history; Identification of cardiovascular risk factors; Use of β-blockers, ACE inhibitors, angiotensin II receptor blockers, apremilast (PDE4 inhibitor), metformin, PKG activator	[201,203,204,205,207,221,224,225,226,227,228]
**Carfilzomib**	Irreversible inhibitor of β5 and β5i subunits	Phase III trial (ASPIRE trial) Prospective, observational study (PROTECT trial)	Relapsed and refractory multiple myeloma	Arrhythmias, heart failure, cardiomyopathy, ischemic heart disease		[212,217,221,224,225,226,227,228]
**Ixazomib**	Reversible inhibitor of β5 and β5i subunits, inhibition of β1 and β2 subunits at high concentration	Randomized phase III trial (TOURMALINE-MM1 trial)	Relapsed and refractory multiple myeloma	Heart failure		[220,221,224,225,226,227,228,229]

## Data Availability

Not applicable.

## Abbreviations

AMPK	AMP-activated protein kinase
ANTs	anthracyclines
ARE	antioxidant response element
BCL-2	B cell lymphoma-2
CHF	chronic heart failure
CMDL-1	cardiomyocyte mitochondrial dynamic-related lncRNA 1
CML	chronic myelogenous leukemia
CVAEs	cardiovascular adverse events
CVDs	cardiovascular diseases
DOX	doxorubicin
Drp1	dynamin-related protein 1
ER	endoplasmic reticulum
Fis1	fission protein 1
FoxO	Forkhead box subgroup O
GPER	G-protein coupled receptor 30
GSH	glutathione
hiPSC-CM	human-induced pluripotent stem cell-derived cardiomyocytes
I/R	ischemia/reperfusion
IMM	inner mitochondrial membrane
Keap1	kelch-like ECH-associated protein 1
LIR	LC-3-interacting region
MAD	mitochondria-associated degradation
MFF	mitochondrial fission factor
MFN1	mitofusin-1
MFN2	mitofusin 2
Midivi-1	mitochondrial division inhibitor-1
MM	multiple myeloma
mtDNA	mitochondrial DNA
NFAT	nuclear factor of activated T-cells
Nfe2l2	nuclear factor erythroid derived 2 like 2
Nrf2	nuclear factor erythroid 2–related factor 2
OMM	outer mitochondrial membrane
OPA1	optic atrophy 1
PDK	pyruvate dehydrogenase kinase
PGC-1α	peroxisome proliferator-activated receptor gamma coactivator 1-α
PGC-1β	peroxisome proliferator-activated receptor gamma coactivator 1-β
Ph+ B-ALL	Philadelphia chromosome-positive B-acute lymphoblastic leukemia
PINK1	PTEN-induced putative kinase 1
PIs	proteasome inhibitors
RNS	reactive nitrogen species
ROS	reactive oxygen species
RTKIs	receptor tyrosine kinase inhibitors
RTKs	Receptor tyrosine kinases
SOD	superoxide dismutase
Top	topoisomerase
TXNRD	thioredoxin reductase
UBD	ubiquitin binding domain
UCP-2	uncoupling protein 2
UCP-3	uncoupling protein 3
UPS	ubiquitin–proteasome system
URPmt	mitochondrial unfolded protein response
VEGF	vascular endothelial growth factor
